# How Prefrail Older People Living Alone Perceive Information and Communications Technology and What They Would Ask a Robot for: Qualitative Study

**DOI:** 10.2196/13228

**Published:** 2019-08-06

**Authors:** Katia Daniele, Maura Marcucci, Cesarina Cattaneo, Nunzio Alberto Borghese, Lucia Zannini

**Affiliations:** 1 Department of Computer Science University of Milan Milan Italy; 2 Geriatric Unit Fondazione IRCCS Ca’ Granda Ospedale Maggiore Policlinico Milan Italy; 3 Department of Biomedical Sciences for Health University of Milan Milan Italy; 4 Department of Health Research Methods, Evidence, and Impact McMaster University Hamilton, ON Canada

**Keywords:** frail elders, independent living, attitude, technology, information technology, robotics, qualitative research, interview

## Abstract

**Background:**

In the last decade, the family system has changed significantly. Although in the past, older people used to live with their children, nowadays, they cannot always depend on assistance of their relatives. Many older people wish to remain as independent as possible while remaining in their homes, even when living alone. To do so, there are many tasks that they must perform to maintain their independence in everyday life, and above all, their well-being. Information and communications technology (ICT), particularly robotics and domotics, could play a pivotal role in aging, especially in contemporary society, where relatives are not always able to accurately and constantly assist the older person.

**Objective:**

The aim of this study was to understand the needs, preferences, and views on ICT of some prefrail older people who live alone. In particular, we wanted to explore their attitude toward a hypothetical caregiver robot and the functions they would ask for.

**Methods:**

We designed a qualitative study based on an interpretative phenomenological approach. A total of 50 potential participants were purposively recruited in a big town in Northern Italy and were administered the Fried scale (to assess the participants’ frailty) and the Mini-Mental State Examination (to evaluate the older person’s capacity to comprehend the interview questions). In total, 25 prefrail older people who lived alone participated in an individual semistructured interview, lasting approximately 45 min each. Overall, 3 researchers independently analyzed the interviews transcripts, identifying meaning units, which were later grouped in clustering of themes, and finally in emergent themes. Constant triangulation among researchers and their reflective attitude assured trustiness.

**Results:**

From this study, it emerged that a number of interviewees who were currently using ICT (ie, smartphones) did not own a computer in the past, or did not receive higher education, or were not all young older people (aged 65-74 years). Furthermore, we found that among the older people who described their relationship with ICT as negative, many used it in everyday life. Referring to robotics, the interviewees appeared quite open-minded. In particular, robots were considered suitable for housekeeping, for monitoring older people’s health and accidental falls, and for entertainment.

**Conclusions:**

Older people’s use and attitudes toward ICT does not always seem to be related to previous experiences with technological devices, higher education, or lower age. Furthermore, many participants in this study were able to use ICT, even if they did not always acknowledge it. Moreover, many interviewees appeared to be open-minded toward technological devices, even toward robots. Therefore, proposing new advanced technology to a group of prefrail people, who are self-sufficient and can live alone at home, seems to be feasible.

## Introduction

### Background

Aging is associated with physiological decay, higher risk of multiple acute and chronic diseases, and ultimately, loss of independence and disability [1]. The constant growth of the proportion of populations represented by older people is challenging the civil society and the health and social systems [1-4]. In this scenario, preventive actions aiming at promoting an active and healthy aging, as opposed to treatment, have the largest potential to reduce the societal burden associated with population aging.

From the individual perspective, many older people want to remain as independent as possible and to remain in their own home; aging in place rather than in nursing home, even when they live alone, is an essential part of this wish [1,4-6]. Aging in place is often considered a good alternative to expensive institutional care by policy makers [6]. Furthermore, “[...] research has shown that there may be significant benefits from delaying or avoiding moving to skilled nursing residences” [5]. However, living alone in older age does not necessarily mean autonomy; rather, it might simply reflect the changes in the structure of the contemporary society, especially in cities, in which children more often move definitively from the nuclear family. In fact, older people living alone are often already in an initial state of vulnerability (prefrail condition), while to safely maintain their independence in their home, one should be able to perform at least some instrumental and basic activities of daily living [1]. Moreover, having a sufficient level of functioning does not necessarily imply satisfaction and well-being, which might also depend on the ability and opportunity to enjoy a social life [7].

There is a growing interest in exploring the potential of information and communications technology (ICT) in interventions to assist frail older people and also to promote active and healthy aging [1,3,6-11]. However, it is commonly agreed that to increase the success of ICT-based solutions, these technologies must be designed according to the end users’ needs. This is particularly relevant, and also challenging, when the user is an older person [3,7,9,12,13]. Their opinion and needs become crucial to plan technological devices, which need to be considered from the beginning of their development. Pursuing the acceptance and adaptation of the older user to an already designed technology has a higher chance of failure [5]. This is one of the reasons why there has been an increased interest among researchers in exploring older people’s perception of technologies. Studies conducted so far have found that different factors might affect older people’s aptitude and attitude toward ICT-based solutions, including psychological and physical condition, personality, life history, culture, and socioeconomic status.

Studies conducted so far have often showed age as a limiting factor for the use of ICT devices [14,15]. Other factors have been also identified as predictors or determinants of technology use among older people, such as education, mood, culture, motivation [2,9,10,14,15], previous experience with technology, ease of use, perceived utility [5,8,11,12,15-17], and the social environment [5,6,8,10,14,16,17].

To our knowledge, no study conducted to explore older people’s preferences on ICT-based solutions to promote independent living has so far focused on a prefrail older population, who might not apparently require assistance for daily activities but, being at risk of deterioration, might still benefit from a support to an independent living. Moreover, we could not find studies looking at the relationship between older people and technologies, conducted in Italy or with participants belonging to a Latin culture. In this sociocultural reality, *familism* —which denotes the centrality of family in the life of people and expectations of mutual emotional and instrumental support among family members throughout the life span [18]—can generate resistance to ICT-based solutions to assist the older person. It is commonly agreed that the users’ cultural background impacts the views and expectations toward technological devices.

### Objective

With this background, we conducted a qualitative study aimed at answering the following question: what are the needs, wishes, preferences, and views about technologies, in general and as a support to independent living, in a sample of Italian prefrail older people who live alone?

This study was part of a wider European project, HORIZON 2020 number 732158, named MoveCare (Multiple-actors Virtual Empathic Caregiver for the Elder), which aims at supporting the independent living of the elderly at home.

## Methods

### Study Design

Assuming that older persons’ attitudes and preferences regarding a complex phenomenon such as ICT are mediated by past events and lived experience with technology, a qualitative study based on the interpretative phenomenological approach (IPA) [19] was designed. This method allows exploring people’s lived experience about a specific phenomenon.

Data were gathered through individual semistructured interviews which, compared with focus groups or group interviews, we believed would have a higher chance of making the older interviewees at their ease, especially when discussing personal issues. This data collection method was largely used in previous qualitative studies based on IPA and on similar topics [4,8,10,14,17,20].

This study has been approved by the Ethical Committee of *Fondazione Istituto di Ricovero e Cura a Carattere Scientifico Ca’ Granda Ospedale Maggiore Policlinico* of Milan, on May 17, 2017. All the participants signed an informed consent containing clear and standardized information about study aims and procedures.

### Sampling

This study was conducted in Milan, Italy.

First, we identified a set of 50 potential participants according to a purposeful sampling. We looked for older people who, at first approach, were willing to talk about their habits and opinions. Moreover, candidates had to satisfy the following criteria:

- age ≥65 years
- living alone (or living alone and receiving assistance for a maximum of 1 hour a day)
- speaking Italian fluently.

From February to May 2017, we sampled members and users of an association of older volunteers named *Associazione Nazionale Tutte le Età Attive per la Solidarietà (ANTEAS;* n=32), people identified through the social work of the municipality of Milan (n=13), patients and relatives referred to the geriatric outpatient clinics of the hospital Policlinico of Milan (n=3), and members of the Italian Union of Retired People of Milan (n=2). The 50 potential participants were contacted by phone. Overall, 4 declined because of health problems and 5 gave no explanation, 4 did not reply to the phone call, 1 did not show up at the appointment, and 1 was not fluent in Italian. The remaining 35 persons were then invited for an interview.

### Procedure

Before the interview, phenotype frailty assessment [21] and Mini-Mental State Examination (MMSE) [22] were administered. To be included in the study, the potential participants had to meet 1 or 2 Fried criteria (ie, prefrail category) and score ≥26 at the MMSE (ie, excluding people with clinically relevant cognitive impairment).

When a potential participant satisfied those inclusion criteria, the full interview was conducted. We drafted an initial interview grid, which in the first part of the interview included questions on the participants’ sociodemographic characteristics, social condition and lifestyle, relationships with relatives and friends, and current health problems and therapies. To explore the experience and attitude toward ICT, the interviewee was asked to imagine a new technology (ie, *like a robot*) available at home and to describe the functions he/she would have asked for it to have. The interviewees were then shown a short video in which an aged woman talked about her experience with Giraff, a previous version of a robot implemented in the MoveCare project [23]. Video was shown from the beginning to 1-min [24,25]. At the end of the video, we again posed the question on the desired functions of the technology, also trying to explore the interviewee’s feelings toward the opportunities offered by a service robot at home. Finally, we investigated the participants’ level of education and asked the interviewees for the income they thought was necessary to have a satisfactory quality of life in their (urban) context. Even though our interview guide was not theory-driven, we considered some studies [1,4,6,8] when formulating some questions of our grid, which was slightly modified after 2 test interviews.

When a candidate did not satisfy the inclusion criteria, we completed a *courtesy interview* on some neutral topics concerning everyday life.

After analyzing the 25 transcriptions, the researchers agreed that data saturation was reached and no more interviews were scheduled. Richards and Morse describe the process of data saturation as follows: “When data offer no new direction, no new question, then there is no need to sample further – the account satisfies. Often, the first sign is that investigator has a sense of having heard or seen it all” [26].

After 2 test interviews, minor modifications were done to the interview grid. The final list of questions is reported in Multimedia Appendix 1. Each interview was performed by one of the 3 investigators involved, who did not have any previous personal or professional relationship with the participants. To guarantee homogeneity and adherence to standards among the interviewers, the senior investigator conducted the test interviews, with the other 2 researchers attending as observers. After the interview and verbatim transcription, the 3 investigators met for debriefing.

Interviews took place in a quiet room in the Policlinico, or at the participant’s home when it was difficult for the older person to travel to the hospital. Each interview lasted about 45 min. At the end of the interview, and later, during its analysis, the researchers wrote memos on what happened during and after the interview and all the reflections that could have been relevant to data analysis [19].

### Analysis

#### Demographics

Sociodemographic and clinical data were extracted from the interviews and processed to convert descriptive/qualitative information into semantic clusters based on the literature and with the help of a geriatrician. For example, a diet was considered *adequate* if the older person declared to have 3 quite balanced meals a day; it was judged *partially adequate* if the interviewee stated to have less than 3 meals a day or 3 meals a day but unbalanced. The diet was considered *inadequate* if the participant declared to have less than 3 meals a day and those meals resulted to be unbalanced. The final classification is shown in Multimedia Appendices 2 and 3. Finally, descriptive statistics were used to report the characteristics of our sample.

#### Qualitative Analyses

Data analysis began as soon as each interview was completed. A total of 3 researchers (with psychopedagogical and/or care background) independently performed the analysis of the interviews. Each interview, transcribed verbatim, was read several times to grasp its global meaning. The coding was data driven. Indeed, each researcher independently identified the meaning units (named in other qualitative methods codes/labels), which were later grouped into clustering of themes. These clustering of themes were later clustered into emergent themes, which were named according to their content. As the clustering of themes emerged, the researchers went back to the transcription to verify its adherence to the participant’s words. The emergent themes were finally related to each other to create a meaningful network. The researchers then discussed each phase of the process until an agreement was reached and the results were reconciled.

## Results

### Participants

A total of 26 candidates eventually met the inclusion criteria. After the full interview, one of the participants retired his/her consent to participate in the study for personal reasons and the file of interview was destroyed.

The characteristics of the 25 participants are summarized in Tables 1-3.

**Table 1:** Participants’ demographic data (N=25).

Characteristic		Values
Age (years), mean (SD)		77.5 (6.6)
**Age (years), n (%)**		
	65-69	4 (16)
	70-74	5 (20)
	75-79	6 (24)
	80-84	6 (24)
	85-89	3 (12)
	90-94	1 (4)
**Gender, n (%)**		
	Male	6 (24)
	Female	19 (76)
**Nationality, n (%)**		
	Italian	23 (92)
	Egyptian	1 (4)
	Austrian	1 (4)
**Education, n (%)**		
	Primary school	4 (16)
	Lower secondary school	10 (40)
	Upper secondary school (ie, high school)	5 (20)
	University	6 (24)

**Table 2:** Participants’ clinical screening data (N=25).

Clinical screening scale		Values
Mini-Mental State Examination, mean (SD)		28 (1.3)
**Fried scale, n (%)**		
	1	13 (52)
	2	12 (48)

**Table 3:** Participants’ quality of life data (N=25).

Variable		n (%)
**Aids and prostheses**		
	None	2 (8)
	Hearing aid	3 (12)
	Cane	4 (16)
	Glasses	22 (88)
**Autonomy in managing money**		
	Partially dependent	6 (24)
	Independent	17 (68)
	Not available	2 (8)
**Autonomy in shopping**		
	Dependent	2 (8)
	Partially dependent	10 (40)
	Independent	13 (52)
**Diet**		
	Inadequate	1 (4)
	Partially adequate	6 (24)
	Adequate	18 (72)
**Help for housekeeping**		
	Never	7 (28)
	Occasional	6 (24)
	Routinely but minimal	5 (20)
	Frequent	4 (16)
	Very frequent	3 (12)
**Medicines**		
	No	3 (12)
	Yes	22 (88)
**Physical activity**		
	None	1 (4)
	Weak	10 (40)
	Moderate	9 (36)
	Good or high	5 (20)
**Quality of sleeping**		
	Bad	8 (32)
	Fair	10 (40)
	Good or high	6 (24)
**Social life—level of engagement in social activities**		
	Weak	3 (12)
	Moderate	8 (32)
	Good or high	14 (56)
**Supposed monthly income**		
	≤€1000	4 (16)
	€1000 to €2000	18 (72)
	≥€2000	3 (12)

### Qualitative Results

As part of the qualitative analysis of the interviews, 477 meaningful units were identified and then grouped into 110 clustering of themes. From these, 13 emergent themes resulted. Overall, 9 of these clustering of themes were related to the quality of life in prefrail older people and will be presented elsewhere. Furthermore, 4 emergent themes were concerning the older people’s attitudes toward ICT-based home services. These were (1) previous and current experiences with ICT, (2) participants’ views of their own relationship with technologies, (3) functions they would ask an imaginary robot for, and (4) sensitivity of the older person’s opinion to others’ experiences with existing ICT solutions for independent living (see Multimedia Appendix 4).

#### Previous and Current Experiences With Information and Communications Technology Devices: Between Continuity and Discontinuity

A clear digital divide was identified among participants in relation to the current use of technologies. One group (about one-third of the participants) declared to use an old mobile phone (number of participants=6) or the landline phone and no other technological devices (n=1); some affirmed they only used mobile phones during holidays (n=2).

The other group (about two-thirds) stated to use 1 or more ICT devices, such as a smartphone (n=13), a computer (n=9), and a tablet (n=1). Among smartphone owners, 3 also owned an old mobile phone. Furthermore, 7 participants affirmed they used WhatsApp. Those who had a smartphone also used some functions/apps such as the camera (n=6), the alarm clock, the calculator, and the memo. Moreover, among the smartphone/tablet owners, 1 used Skype and 2 used Facebook. Those who owned a smartphone (or a computer) used it to play Web (eg, Burako, a card game; n=3) or not Web-based (solitaire; n=1). In addition, 5 smartphone owners declared that they had an internet connection but were unable to use it at that time. A total of 3 participants declared that they had some difficulties in using the touch function of their smartphone; however, 2 of them affirmed that they could find some strategies to overcome those difficulties.

Technological devices were mainly used by participants to communicate and interact with relatives or friends. To this purpose, some informants specified that they used the smartphone to make phone calls (n=4), for emails (n=1), and for the app WhatsApp (n=1). The computer (internet) was used for email (n=8), video calls via Skype (n=4), to receive news from relatives (n=3), and to receive photos (n=2).

One interviewee (Interviewee 12) said she had an emergency call device, but she never used it as she was afraid of making unintentional calls that would have alarmed her caregivers unnecessarily.

Technological devices were not only used by the participants for communication purposes; internet was also used to search for recipes (n=1), for home banking (n=3), to search for the meanings of new words, or to look for information (n=7):

[...] Google Earth, that map used for searching Geography. [...] Well yes, [I search] any information, from Geography to historical figures, some historical figures, some art works

Interviewee 9

Moreover, the participants declared that they used computers for some applications such as Office (n=3; “Of course, [I know] how to write documents using Word, working on tables...”—Interviewee 5), for their personal accountancy (n=1; “I use it to manage the household accounts”—Interviewee 5), to archive photos (n=1), and to watch Digital Versatile Discs (n=1).

Furthermore, from the analysis of the interviews, 3 trends emerged concerning the current use of ICT in relation with the past use of ICT, that is, at a younger age. About one-third of the participants used a computer in the past (both for work and for personal interests) and still used either a computer (n=6) or a smartphone (n=6). Most had an internet connection. An exception in this group was represented by 1 interviewee who used a computer in the past, but at the time of the interview, the only ICT he/she owned was an old mobile phone.

A second trend was represented by those (about one-third of the participants) who did not use a computer in the past and just used an old mobile phone at the time of the study.

Finally, a third group of participants did not use a computer in the past, but declared to currently use 1 or more ICT devices, such as a smartphone (n=7), a computer (n=3), or a tablet (n=1).

With regard to the association with the level of education, about half of the participants who had a computer in the past, received a postsecondary level of education. Conversely, among the 13 smartphone owners, only one-third went through higher education. Surprisingly, we did not find any obvious association between the participants’ age and their use of technology.

#### Older People’s Views of Their Relationship With Information and Communications Technology: Thinking Negatively, Acting Positively

Almost one-third of the interviewees (n=11) did not have an opinion about their relationship with technological devices. One-third (n=9) claimed to have a negative relationship with ICT, that is, endorsing a feeling of denial, inadequacy, or a lack of expertise:

[...] The only thing...because I’m not good at these technological things

Interviewee 2

My relationship with technologies is very bad, very bad, very bad. [...] I think I refuse [to learn] it, because I do not think I’m so stupid not to be able to

Interviewee 6

For goodness sake, It’s already so much that I’ve used this [the phone]! Absolutely! I’m not able to use them [technological devices].

Interviewee 7

This type of feeling was not necessarily associated with a refusal to use ICT. In fact, 8 of those who considered their relationship with technologies as negative (n=11) used several devices in their daily life (ie, smartphones and computers).

A total of 8 participants declared that they needed, and someway sought for, some help to be able to use ICT:

Yes, but, for the most tremendous things, my brother in law used to come, he was the king of technology.

Interviewee 6

Then, if a payment via home banking has to be completed, well...it’s made by my son or my daughter.

Interviewee 9

In addition, 5 of those who could not define their relationship with ICT actually used several devices.

A small group of participants affirmed that they were in favor of new technologies (n=1), or stated that they wanted to improve their relationship with ICT or they wanted to adapt themselves to “advancing technology” (Interviewee 8), for example, by learning how to use their smartphone better (n=2), or by attending an informatics course (n=1). One participant said he/she wanted “to live in the technological era” (Interviewee 11).

Finally, 3 participants clearly stated that ICT was not a fundamental part of their lives, as “you can work the same without having a computer” (Interviewee 7) or was not “essential” (Interviewee 16). One interviewee declared he/she still used a paper telephone book “only because I often lose my cell phone” (Interviewee 15). However, 2 of these participants actually used a computer (Interviewee 15) or a smartphone (Interviewee 16).

#### Functions That Older People Would Ask a Hypothetical Robot for: Between Housekeeping and Need for Company

Most of the participants (n=18) wished to own a robot that helped them with cleaning the house. According to an interviewee, a robot could be a valid substitute of the housekeeper:

[A small robot should] first of all, do the cleaning and then I don’t know, nothing else, because I’m only interested in having the house cleaned and tidied up.

Interviewee 1

Among these 18 interviewees, only 7 did not have any help with housekeeping activities.

Cooking was another desired task (n=5). One participant imagined that the robot could even be used as an oven.

Some participants (4) asked for a multitasking robot, able to act as a formal caregiver or babysitter or maid. According to 1 participant, it might be a machine with “its own intelligence” (Interviewee 7), able to perform the tasks that a human does not want to do or cannot do. Following this request of a *multitasking robot*, 1 participant imagined that this mechanical device could be able to drive a car. Another participant imagined the robot to go shopping with him. Others (n=2) wanted this hypothetical robot to go shopping for them or to buy their medicines, if they were unable to go by themselves (n=2). One participant asked for help with getting washed and dressed. Finally, 1 participant asked for support with physiotherapy.

Other participants (n=3) believed that a robot should be able to monitor the old person and ask for help in case of emergency:

If I fall, I fall. I can’t get up and knock at my neighbour’s door. And if...if I had the robot instead...very good [...] If the robot is present, it doesn’t sleep, it hears that something doesn’t work, or it rather has a sensor which tells it directly and it...does what it has to...nips, calls 113, I don’t know, 118 [emergency telephone numbers], it does anything [...] well [I would like] it to monitor me...and that it should intervene in case I couldn’t make it.

Interviewee 2

On the other hand, about one-third of the participants would ask for a robot with more entertaining functions. The imaginary robot could itself be a companion (n=3) to listen to music (n=1), sing together (n=1), play cards (n=1), or share hobbies with (n=1). It was otherwise seen as a provider of general leisure activities (n=1), or to keep abreast of news (n=1), or as a tool to learn writing and reading (n=1). Most of the interviewees in this second group had a *weak* or *fair* social life.

Finally, a small group of participants (n=3) claimed that a robot could not only be useless but also a deterrent for those people who can perform some tasks autonomously (eg, housekeeping).

#### Sensitivity of the Old Persons’ Opinion to Others’ Experiences With Existing Information and Communications Technology Solution for Independent Living

After watching the video about Giraff, a robot carer which offers company and some monitoring functions [23], the participants’ opinions on ICT undertook a substantial change.

The most requested function from a robot was still related to cleaning the house, but this request was expressed by 9 participants instead of 19.

The second most frequent request, after watching the video, became the robot’s ability to ask for help in case of emergency (n=9), falls (n=1), and danger, in general (n=1). In particular, according to 5 participants, the robot could be useful in case of gas leaks, open windows, or an attempted break-in (n=5).

Moreover, only after watching the video, the participants could endorse the usefulness of the robot in monitoring vital signs (n=4). One person even spoke about “being monitored 365 days per year” (Interviewee 6). Giraff was not only perceived by some participants (n=3) as a useful instrument to remind the older person to perform some health-related tasks (eg, measuring their blood pressure and taking pills), but also as a reminder for buying groceries. The function of preparing meals was still considered important by 2 participants.

The robot was also acknowledged, but only minimally, as a communication instrument (n=2); indeed, it was considered by 1 participant as a valid alternative to a computer or a tablet. Moreover, an informant speculated an amplification function for people who suffer from hearing problems.

However, the idea that a robot could be a companion for an older person increased after watching the video (mentioned by 7 participants). One participant sustained that Giraff could be useful to go for walks together, and another imagined it could drive a car and take a person out. The fact that the robot could not “speak, offend, or get angry like a human being” was mentioned as an advantage of this special companion by 1 participant (Interviewee 9).

Some participants considered Giraff as a useful caregiver for those who are in old age (n=4) or have cognitive problems. However, although some informants still endorsed that the robot could perform important assistance functions in the case of people with lower levels of autonomy, overall, after watching the video, the participants tended to scale down the robot’s functions compared with the multitasking robot they had imagined before.

Interestingly, Giraff was seen as inadequate if an old person was self-sufficient in performing certain tasks (n=4) or even as a deterrent to keep oneself active and self-sufficient (n=2):

I don’t know, these things...all those possibilities, it’s true, they exist and in my opinion they are very good for those who, unfortunately, can’t move and then I can understand, but, as long as a person is self-sufficient and can move freely, I think they are wasted potentialities.

Interviewee 4

Yes, well it does anything so, then...I don’t close the window anymore, I don’t switch off the light, it turns the light on, it does anything! I stay there completely still...so, O.K. when I am 102 years old...I agree...But if it does these things now using technology...when it gets dark all the lights go on, there are lots of things around...well, human beings don’t do anything anymore! [Technologies] can do all these things, if the door or the window are open, they close them...so I sit on my chair or in an armchair with that thing opposite me and I don’t move anymore.

Interviewee 8

One participant highlighted that Giraff cannot be useful in increasing social relationships, particularly for self-sufficient people. Another participant did not consider this robot as a valid alternative or as an addition to other devices such as computer and telephone. One participant stressed the fact that Giraff could be annoying for those who live in a small house.

We noticed that the participants’ capacity to change their mind (about the possible functions of the robot), after watching the Giraff video, were mainly related to their baseline relationship with ICT, that is, the more positive it was, the more easily they changed their mind and could grasp the potentialities of a robot aimed at assisting an older person who lives alone.

## Discussion

### Principal Findings

Our study partially challenges the previously reported positive association between older people’s past and current use of ICT, as well as the positive association between the current use of ICT and higher education and lower age [4,12,14,15]. From our study, it emerged indeed that a number of interviewees who were currently using ICT (ie, smartphone) did not own a computer in the past, or did not receive higher education, or were not all *young* *older people* (aged 64-75 years). Furthermore, we found that a negative view of ICT (thinking negatively) not always corresponds to its actual rejection or underuse in everyday life.

Referring to robotics, our interviewees appeared quite open-minded. In particular, robots were considered suitable for housekeeping, for monitoring older people’s health and accidental falls, and for entertainment. After watching the Giraff video [23], many interviewees acknowledged those functions and even glimpsed other potentialities.

Although ICT is providing a wealth of new devices and possibilities, because of the common belief that older people are not keen to adapt or use new technologies in the same way younger generations would, researchers have been very cautious in developing new platforms, applications, and systems for older people [27].

Indeed, literature reported some correlation between the positive or negative perceptions of ICT and older people’s level of education, age, and previous familiarity with technological devices [4,12,14,15], thus supporting the theory of an educationally based digital divide. However, according to our findings, being open-minded to technologies seems to be only loosely related to older people’s level of education, contrary to what was reported by other studies [14]. In our study, just half of the participants who had a computer owned a degree, and among smartphone owners, just one-third had received higher education. Furthermore, we did not find correspondence between older people’s lower age and greater use of technology, contrary to previous studies [14,15]. In accordance with the existing research [4,12], we found that the actual use of technology seems more correlated with the previous use of technological tools. We also found that those who used a computer in the past seemed to be more open-minded toward new technologies, particularly smartphones. However, having used technology in the past does not seem a necessary condition to accept technology in the older age [15]; even if some participants in our study did not own a computer in the past, they did currently have a smartphone and were able to use it, not only to communicate, but also for other functions offered by the device. This is quite an important finding and it may be explained by the fact that today’s technologies are certainly more available and immediate than in the past, and therefore more accessible to older people too, with little adaptation. This process seems to improve older people’s digital literacy. This is confirmed by the fact that previous familiarity with technology seems to have a greater impact on the current use of computers and less on smartphones and tablets use, whose interfacing modality is more intuitive [28].

Concerning the actual use of technological devices, we found that they are used by more *technological* older people for several aims. The most frequent use is to communicate with their children and relatives, but also to search for the meaning of some words, recipes, and to manage their money (home banking).

In accordance with a recent study [6], our results indicate that the greatest impact on the use of technologies is determined by their views: the more positive it was, more participants said they used technologies with satisfaction. However, the relationship between technologies views and their use is not straightforward. In fact, we detected some ambivalence in the participants’ words. A conflicting position sometimes emerged from the interviews about the object (the technologies that the participants actually used) and the way older people represent technologies to themselves. We discovered that even among the participants who claimed to have a negative relationship with technologies, some familiarity with them can be found in practice. It seems that negative views of technology do not always affect their actual use; on the contrary, sometimes older people tend to think negatively about technology, but act positively toward it.

In general, the older people in our study had many negative preconceptions about technology. They often declared that they were not able to use it despite the fact that they actually used several devices in their daily life. This feeling of inadequacy or incompetence in using technologies also emerged from other studies [8-10], and it seems to be reflected in our interviewees’ need to receive some help when using technologies. Therefore, providing technical support appears to be fundamental when proposing new technologies to older people.

With regard to the functions that the participants would ask a hypothetical robot for, when assisting them at home, we found that they require, above all, cleaning functions. In some cases, they asked for support in preparing meals and in other cases they requested to have their health state monitored or to receive monitoring and aid on request, as already reported in literature [1,7]. From the analysis of the participants’ requests, it seems that the desire to maintain autonomy and avoiding the help of a caregiver at home, especially for cleaning, could make them accept a robot in their home, as reported by existing literature [4,17,29]. Those participants who claimed for a multitasking robot seemed to be moved by the same reasons, although simultaneously they made an unrealizable request. It could be interpreted as a rejection of a robot at home or conversely as an unexpressed request for human aid, which can effectively act as a maid. A few participants stated that a robot could be useless or discouraging for those people who could still perform some tasks and therefore have a negative impact on their health and autonomy.

Health status is a common concern of all participants. A vital parameter control function was also considered important by participants, especially for the anxiolytic effect it may have on them. They also requested an option for Web-based shopping (although some have pointed out that the everyday shopping is important for the older people’s health) and functions that are similar to tools such as the *Bimby or Thermomix*, a kitchen robot which helps to prepare meals.

The need of sociality also emerged clearly. Some older people (especially those who stated that they had a poor or average social life) would ask a hypothetical robot for entertainment-related functions such as listening to music, singing, playing cards, receiving information or keeping up-to-date, and even sharing hobbies. A robot supporting older people living alone could thus have management and monitoring functions, as well as entertainment and company functions.

After watching the Giraff video, many of the above functions advocated by participants were confirmed, some reinforced, and new possibilities on socialization envisaged. The interviewed participants understood more clearly the usefulness of a robot in monitoring and managing emergencies, particularly the Giraff’s function of calling in case of emergency, which was acknowledged by one-third of the participants. One-third of the participants highlighted the perceived usefulness of Giraff in detecting gas leaks or intrusion of strangers into the house. From the socialization point of view, the participants’ acknowledgment of a robot’s ability to communicate and entertain increased after watching the video. This is an interesting aspect as people may not be automatically disposed to recognize a nonhuman interlocutor (the robot) as a converser in communication processes or as a leisure companion. One participant even emphasized that the robot “could not speak, offend, and get angry like a human being”; so, this mechanical device could be sometimes preferred to the company of a person.

We found that although only a few participants considered that a robot with the abovementioned functions would be useful to those who lost their autonomy, many realized that such a device would help maintain one’s own autonomy and be able to keep living alone in their home.

The way participants changed their mind (before and after watching the video) on the possible functions of a robot assisting them at home, seems to be correlated to the views of the technologies that the informants had previously expressed: the more positive it was, more they easily changed their mind and could grasp the potentialities of a robot aimed at assisting the older person who lives alone. Therefore, when proposing a robot to a prefrail elder living alone, the relationship with technologies and their views should be carefully evaluated. Both the older person and the other significant ones around them must perceive the usefulness of technologies, for example, in the contribution that they can give to their safety.

The functions that our participants asked a robot for are perfectly in line with those recommended by others belonging to different cultures, such as house cleaning, help in finding information, detecting falls or domestic accidents, monitoring vital parameters, and even in walking around [1,7]. In addition to these functions, more related to house management and to health monitoring, some participants expressed their desire for a company or entertainment function that may counteract a condition of loneliness referred by many older people as indeed a cause of frailty.

In conclusion, the results of our study indicate that a technological device such as Giraff could be well received by prefrail older people who live alone at home, even when they belong to a Latin culture (Mediterranean countries and Latin America), where the elder’s assistance is commonly delegated to family members, usually women. In some cases, the robot was considered useful in communication processes or even as a leisure companion. This means that some participants could consider such a device as a remedy to their loneliness. This does not mean considering a technology such as Giraff as a substitute of a human person, but as a tool to maintain and broaden one’s relationships and interests.

### Limitations

The qualitative design of this study entails both strengths and limitations. Though we were able to examine data not easily accessible to quantitative research, these results are not transferable. An additional limitation stems from our recruitment mostly coming from ANTEAS, in which participants volunteered or participated in recreational activities. Therefore, even if purposively sampled, the majority of our participants could be defined as *socially active older people*. Furthermore, our participants lived in a big city. Different perspectives on technologies could be gathered from prefrail older people who live alone in a countryside.

### Conclusions

This study provides insights on the perceptions of new technologies in a group of prefrail older people. This is a target of potential users that has received little attention by the literature. Our study suggests that many participants were able to use technologies, even if they did not always recognize it. Moreover, many seemed to be open-minded toward technological devices, even toward robots.

Moreover, some older people, even from Latin culture where *familism* —a strong commitment to the family as a system of support, learning, socialization, and assistance—can be a core cultural value, could recognize among the functions that a robot should have, some not trivial ones, such as company and entertainment.

Therefore, proposing new advanced technology to a group of prefrail older people, who are self-sufficient and live alone at home, is both attainable and desirable. However, before proposing robots to this target group, it is highly recommended to conduct a pilot study on the development of such new technology to make it more suitable for the end users, as we began to do in this study.

## Appendix

Multimedia Appendix 1 Interview grid.

Multimedia Appendix 2 Classification of the participant data on function and lifestyle—1.

Multimedia Appendix 3 Classification of the participant data on function and lifestyle—2.

Multimedia Appendix 4 The emergent themes resulting from the older people’s interviews on information and communications technology.

## Abbreviations

ANTEAS: Associazione Nazionale Tutte le Età Attive per la Solidarietà
ICT: information and communications technology
IPA: interpretative phenomenological approach
MMSE: Mini-Mental State Examination
