# Supplementary material for: How Prefrail Older People Living Alone Perceive Information and Communications Technology and What They Would Ask a Robot for: Qualitative Study
Source: J Med Internet Res. 2019 Aug 6;21(8):e13228. doi: 10.2196/13228 (PMC6701159; doi:10.2196/13228)
Supplement: Multimedia Appendix 1 [file jmir_v21i8e13228_app1.pdf]

1. Would you tell me about your typical day?
    a. Do you like cooking? (Explore elder’s diet, what he/she usually has for breakfast, lunch and dinner. Investigate if he/she eats more at lunch or dinner, if he/she has meals alone, if someone cooks for him/her...);
    b. Do you usually take a walk during the day? (Explore the elder’s habits about physical activity, smoking and social relationships);
    c. Are you used to having a nap in the afternoon? (Explore the quality of sleeping, e.g. if he/she sleeps in the afternoon because does not sleep well during the night and why).
2. Do you have any difficulties in your daily life? (Explore the use of aids and prostheses, as glasses, cane, audio-prostheses); How do you manage shopping, money, meds?
3. In which of the above activities do you receive any help? In which of those activities would you like receiving help?
4. What is important in your life now?
5. How are your relationships with family and friends? (Explore level of satisfaction)
6. What do you miss in your life now (making a comparison with past time). What could make your life more satisfying?
7. How would you define your actual health status?
    a. How many meds do you take every day?
    b. Do you go to the doctor’s frequently?
    c. Have you been to a hospital recently?
8. Can you tell me about your habits and aptitude concerning the use of technologies, such as mobile phones and computers? (Explore when the elder started using a certain device, how long the device is used during a day, for which scope, which difficulties the elder encounters in using that device, factors that consented to overcome those difficulties. If the elder has a good relationship with technologies, try to understand what strategies were used)
9. If you won a little robot at a lottery, what would you ask the robot for? (Explore which functions the robot should have for managing daily life, information, therapies, social relationships….).
10. Now I am showing you a video in which a person like you talks about a robot that lives with her, at home (Giraff video).
11. Now, after you watched this video, what would ask a robot for?
12. A few last questions to conclude the interview… Can you tell me about your education?
13. Have you always lived in Milan?
14. A last question, just to have your opinion: how much should be the income of a person like you, who lives at home alone, in order to have an adequate quality of life?
