# Supplementary material for: How Prefrail Older People Living Alone Perceive Information and Communications Technology and What They Would Ask a Robot for: Qualitative Study
Source: J Med Internet Res. 2019 Aug 6;21(8):e13228. doi: 10.2196/13228 (PMC6701159; doi:10.2196/13228)
Supplement: Multimedia Appendix 2 [file jmir_v21i8e13228_app2.pdf]

Variable	Value	Value description
**Autonomy in managing money**		
	**Dependent**	No autonomy in managing money and economic/bank activities and helped by a son/daughter or someone else.
	**Partially dependent**	Autonomy in managing money but she/he receives support for more complex economic/bank activities.
	**Independent**	Complete autonomy in managing money and economic/bank activities.
**Autonomy in shopping**		
	**Dependent**	She/he receives help in shopping/no autonomy.
	**Partially dependent**	She/he receives some help, i.e. doesn't carry heavy things that are taken home by others, or buys by his/her self but uses home delivery services **and/or** she/he receives meals from a service offered by the town hall.
	**Independent**	She/he buys and goes shopping on her/his own.
**Diet**		
	**Inadequate**	Less than 3 meals a day **and** not balanced/poor quality of the diet.
	**Partially adequate**	Less than 3 meals a day with balanced dishes **or** 3 meals but not balanced/poor quality of the diet.
	**Adequate**	3 meals a day **and** a balanced diet.
**Help for housekeeping**		
	**Never**	No help for housekeeping.
	**Occasional**	Once in a while.
	**Routinely but minimal**	One or more times a week for less than 3 hours a week.
	**Frequent**	One or more times a week for less than 7 hours a week but for at least 3 hours a week.
	**Very frequent**	Every day for at least 1 hours a day or not every day but at least 7 hours a week.
