# Supplementary material for: How Prefrail Older People Living Alone Perceive Information and Communications Technology and What They Would Ask a Robot for: Qualitative Study
Source: J Med Internet Res. 2019 Aug 6;21(8):e13228. doi: 10.2196/13228 (PMC6701159; doi:10.2196/13228)
Supplement: Multimedia Appendix 3 [file jmir_v21i8e13228_app3.pdf]

Variable	Value	Value description
**Physical activity (intensity)**		
	**None**	Never exercises and rarely goes out, only for medical examinations
	**Weak**	Very mild physical activity, i.e. sometimes goes for walks, mainly for errands.
	**Moderate**	Sometimes exercises – once a week or less- and/or often goes out and walks at a good pace.
	**Good or high**	Follows regularly an exercise program and she/he goes out every day and walks at a good pace.
**Quality of sleeping**		
	**Bad**	She/he says to sleep badly, for example not much (<=5 hours) and/or with breaks/interruptions.
	**Fair**	She/he says to sleep quite well but for not many hours (<=5) **or** many hours with some breaks/interruptions.
	**Good or high**	She/he says that she/he sleeps well and enough.
**Social life – level of engagement in social activities**		
	**None**	She/he doesn't see/meet anybody.
	**Weak**	She/he sees/meets few people, only relatives/caregivers.
	**Moderate**	She/he sees/meets people, not only relatives or caregivers, but also friends twice a week or less.
	**Good or high**	She/he often goes out for social activities like having dinners, going to the theatre and/or attends circles, associations, parish etc…
**Desired monthly income [emerged from the answers to the "indirect" question number 14 of Interview grid (see Box 1)]**		
		≤1000 €
		1000 € - 2000 €
		≥ 2000 €
