# Supplementary figures and images for: How Prefrail Older People Living Alone Perceive Information and Communications Technology and What They Would Ask a Robot for: Qualitative Study
Source: J Med Internet Res. 2019 Aug 6;21(8):e13228. doi: 10.2196/13228 (PMC6701159; doi:10.2196/13228)

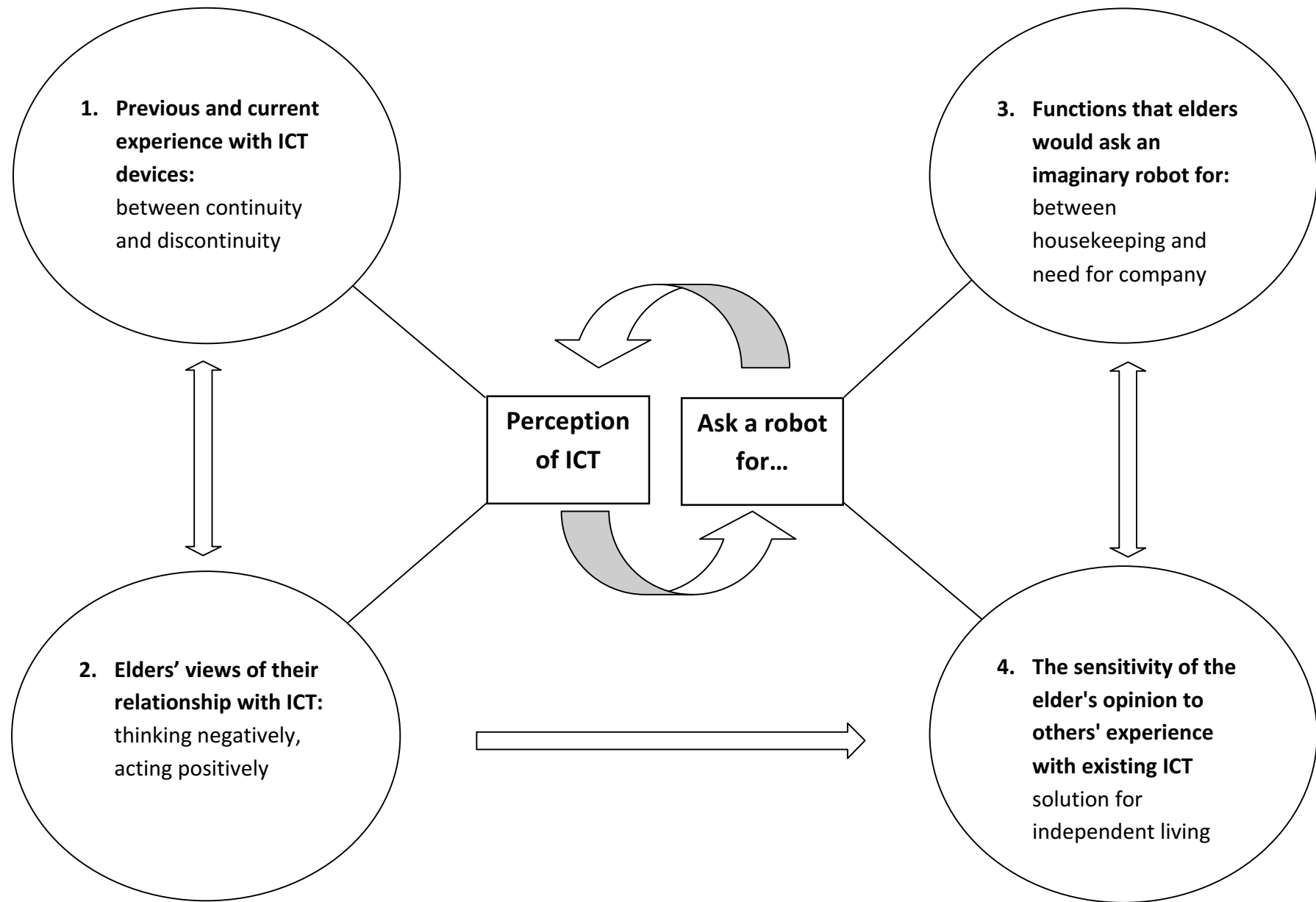

Supplement: Multimedia Appendix 4 [file jmir_v21i8e13228_app4.pdf]
